# Status and trends in transcranial magnetic stimulation research: a bibliometric analysis

**DOI:** 10.3389/fneur.2025.1593987

**Published:** 2025-05-20

**Authors:** Qi Deng, Fei Xing, Dan Li, Maomao Huang, Zhangyu Xv, Yue Yang, Lei Li, Jiayi Zhu, Rongnan Shi, Guoya Meng, Qin Wang, Jianxiong Wang

**Affiliations:** ^1^Rehabilitation Medicine Department, The Affiliated Hospital of Southwest Medical University, Luzhou, Sichuan, China; ^2^Rehabilitation Medicine and Engineering Key Laboratory of Luzhou, Luzhou, Sichuan, China; ^3^Department of Rehabilitation Medicine, Southwest Medical University, Luzhou, Sichuan, China

**Keywords:** transcranial magnetic stimulation, repetitive transcranial magnetic stimulation, bibliometric analysis, bibliometrics, visualization analysis

## Abstract

**Background:**

Transcranial magnetic stimulation (TMS), as a rapidly advancing non-invasive brain stimulation technique, has demonstrated reliable therapeutic efficacy and significant potential in brain function assessment and research. However, comprehensive bibliometric analyses of the overall TMS field remain limited. Therefore, this study conducts a systematic analysis of TMS-related literature from 2004 to 2023, aiming to facilitate further advancements in TMS research and development.

**Methods:**

We retrieved TMS-related literature from 2004 to 2023 in the Web of Science Core Collection. Using CiteSpace and R language, we constructed network visualizations to illustrate annual publication outputs and journal distributions, demonstrate co-occurrence and collaboration patterns among authors, countries, and institutions, and establish keyword co-occurrence and reference co-citation analyses.

**Results:**

Our analysis incorporated 6,278 TMS-related articles. Despite fluctuations, the publication output demonstrated an overall upward trend over the 20-year period. Daskalakis Z.J. emerged as the most prolific author, while the United States and Harvard University were identified as the leading contributing country and institution, respectively. *Brain Stimulation* ranked first in publication volume, whereas *Clinical Neurophysiology* received the highest citation count. The work by Rossi S. achieved the highest co-citation frequency. Current research hotspots include intermittent theta-burst stimulation, cognitive impairment, systematic review, and mild cognitive impairment.

**Conclusion:**

Research related to TMS has been increasing annually and is a developing field. The United States leaded the way, while Harvard University was the most active institution. Daskalakis Z.J. (Canada) was the most prolific author. The most influential journals included *Brain Stimulation*, *Clinical Neurophysiology*, and *Journal of Affective Disorders*. Further deep collaboration among leading countries, institutions, and authors is needed. Current hotspots in TMS research involve integration with imaging techniques, clinical applications, optimization of parameters, and exploration of neurological modulation mechanisms.

## 1 Introduction

Transcranial magnetic stimulation (TMS), as a non-invasive brain stimulation technique, is playing an increasingly important role in clinical diagnosis and treatment with the rapid development of neuroscience and neuromodulation technology. TMS includes Single-pulse TMS (sp-TMS), paired-pulse magnetic stimulation, and repetitive transcranial magnetic stimulation (rTMS), among which rTMS includes High-frequency rTMS (Hf-rTMS) (>1 Hz, enhancing cortical excitability) and Low-frequency rTMS (Lf-rTMS) (<1 Hz, inhibiting cortical excitability) (1).

rTMS has demonstrated promising therapeutic potential in managing severe central nervous system injuries, showing efficacy in ameliorating various dysfunctions such as paralysis, spasticity, speech impairment, cognitive deficits, and pain associated with spinal cord injury, stroke, cerebral palsy, and multiple sclerosis (2, 3). Studies have shown that TMS plays an important role in some psychiatry diseases, such as depression, post-traumatic stress disorder (PTSD), and others (4, 5). Compared with rTMS, theta burst magnetic stimulation (TBS), has similar or better outcomes in treating depression effects (6).

The significance of TMS extends beyond its therapeutic applications, encompassing a pivotal role in brain function research and diagnostic procedures. For example, sp-TMS is a versatile tool and technique for electrophysiological assessments. For instance, it has been utilized to monitor cortical activity changes in patients with Alzheimer’s disease (7). Furthermore, motor evoked potentials (MEP) induced by sp-TMS have been employed to evaluate the effects of cerebellar rTMS on the pharyngeal region of the motor cortex (8). The integration of TMS with electroencephalography, known as TMS-EEG, has emerged as a robust investigative tool. This combined modality facilitates research on the effects of antiepileptic drugs on cortical excitability (9), while also enabling the examination of neuroplastic changes associated with analgesic mechanisms in the dorsolateral prefrontal cortex (DLPFC) (10). This technique enables non-invasive investigation of human brain circuits, allowing for the assessment of cortical properties such as excitability and connectivity (11). The integrated application of TMS and near-infrared spectroscopy (NIRS) greatly achieves precise diagnosis, assessment, and targeted therapeutic intervention for brain functions, especially simultaneously promotes functional rehabilitation in specific areas and activates neural network reorganization (12, 13). TMS technology has also been implemented in the screening of early-stage cognitive dysfunction, localization of epileptogenic zones in refractory epilepsy, and mapping of cortical language and motor functions (14–16). Recent progress in TMS technology and its integrated applications has resulted in numerous research breakthroughs in the field of TMS studies.

Bibliometrics, as a scientific discipline, systematically investigates the literature publication, dissemination, and utilization (17). Yang et al. conducted an analysis of the current research status of TMS in depression (18), focusing exclusively on its therapeutic applications for this condition. Xiao and colleagues reviewed the applications of TMS in autism spectrum disorders from 1992 to 2022 (19). However, new TMS-related studies continue to emerge. Mariana F. G. Lucena et al. conducted a bibliometric analysis on non-invasive neuromodulation, which included TMS (20). However, their study was not specifically dedicated to TMS-related literature and lacked comprehensive co-occurrence analysis of authors, countries, and research institutions.

Therefore, the current bibliometric analysis of the overall field related to TMS remains insufficiently comprehensive. Given the widespread application of TMS technology in clinical practice and the ongoing advancements in understanding its neurophysiological mechanisms, it is essential to conduct both quantitative and qualitative evaluations of the literature related to TMS research. So this paper will analyze TMS-related literature from 2004 to 2023, with the hope of further encouraging researchers to explore the unknown areas of TMS and providing some reference for the future research directions of TMS.

## 2 Method

On September 28, 2024, we conducted a search in the Web of Science Core Collection (WOSCC, index: SCI-EXPANDED) for articles related to TMS research. Our search query was as follows: TI = ((“Repetitive transcranial magnetic stimulation”) OR (“rTMS”) OR (“transcranial magnetic stimulation”) OR (“Theta-burst stimulation”) OR (“Theta burst stimulation”) OR (“triple stimulation technique”) OR (“paired associative stimulation”) OR (“paired bihemispheric stimulation”)). The search was restricted to the period from January 1, 2004, to December 31, 2023. The document types included were articles and review articles, and the language was limited to English.

**Figure 1:**
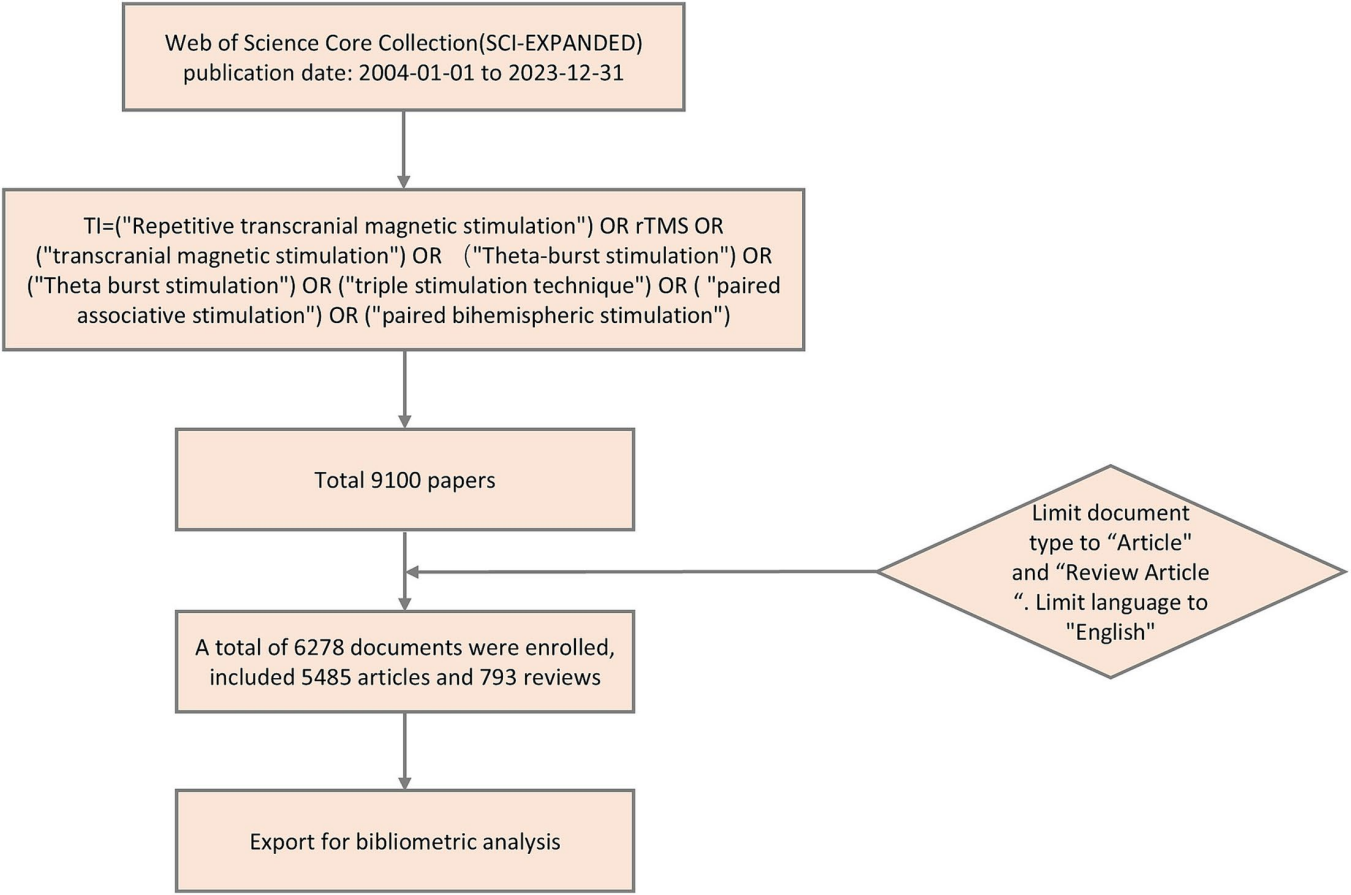
Flowchart of the literature search and analysis process.

Firstly, we conducted a search based on the WOSCC to obtain general information on the annual publications, authors, institutions, countries, journals, and funding sources. Additionally, we employed the H-index to measure the impact of journals or authors (21), which integrates both citation counts and the number of publications. The higher the H-index, the higher the impact. The G-index is utilized to evaluate the academic influence of scholars. Its calculation method involves ranking the author’s published papers in descending order based on the number of citations. The cumulative sum of the squared ranks of each paper is then computed. The G-index is determined when the squared rank is less than or equal to the cumulative number of citations (22). Subsequently, the Citespace software was employed to conduct co-occurrence analyses among authors, institutions, and countries. Thereafter, co-citation analyses of references, authors, and journals were performed using Citespace to elucidate the research foundation in the field of TMS. Finally, to obtain insights into the cutting-edge knowledge and research trends related to TMS, we utilized Citespace and the Bibliometrics package in the R language to perform co-word analysis of keywords and generate thematic maps, respectively. Figure 1 illustrates the workflow of the literature search and analysis process.

The parameters for the Citespace settings are as follows: slice time 2004–2023, 1 year per slice, term source (all selection), node type, and pruning (pathfinder and pruning the merged network). Node types include author, institution, country, cited journal, cited author, reference, and keyword. Node size indicates the frequency of occurrence, and the line between nodes indicates the presence of co-occurrence or co-citation relationship between them, and the width of the line indicates the strength of co-occurrence. Median centrality represents the number of shortest paths through a node in a network and is used to measure the importance of a document. The greater the mediator centrality, the greater the role it is considered to play in the communication between other nodes. When the mediator centrality is greater than or equal to 0.1, we mark it with a purple circle. The width of the annulus is proportional to the number of citations it has; the wider the annulus of a node, the more citations it has at that time. Clustered network analysis was used to analyze references, co-cited authors, and keyword co-words. When the silhouette value is >0.7, the clustering results are considered to have high reliability, the closer the silhouette value is to 1, the higher the homogeneity of the clustered network is, the larger the Q value is, the better the clustering of the network is represented, and when Q > 0.3, the network clustering structure is significant.

The bibliometrix package in R provides robust statistical analyses and graphical techniques, which can clearly and scientifically present collaboration world maps, thematic maps, and the evolution trends of thematic terms. The thematic map is divided into four quadrants: the upper right quadrant represents thematic terms that are important and well-developed; the lower right quadrant represents thematic terms that are important but not well-developed; the upper left quadrant represents thematic terms that are well-developed but not important to the current field; the lower left quadrant represents peripheral thematic terms, which may have just emerged or may disappear.

## 3 Result

### 3.1 Publication outputs

**Figure 2:**
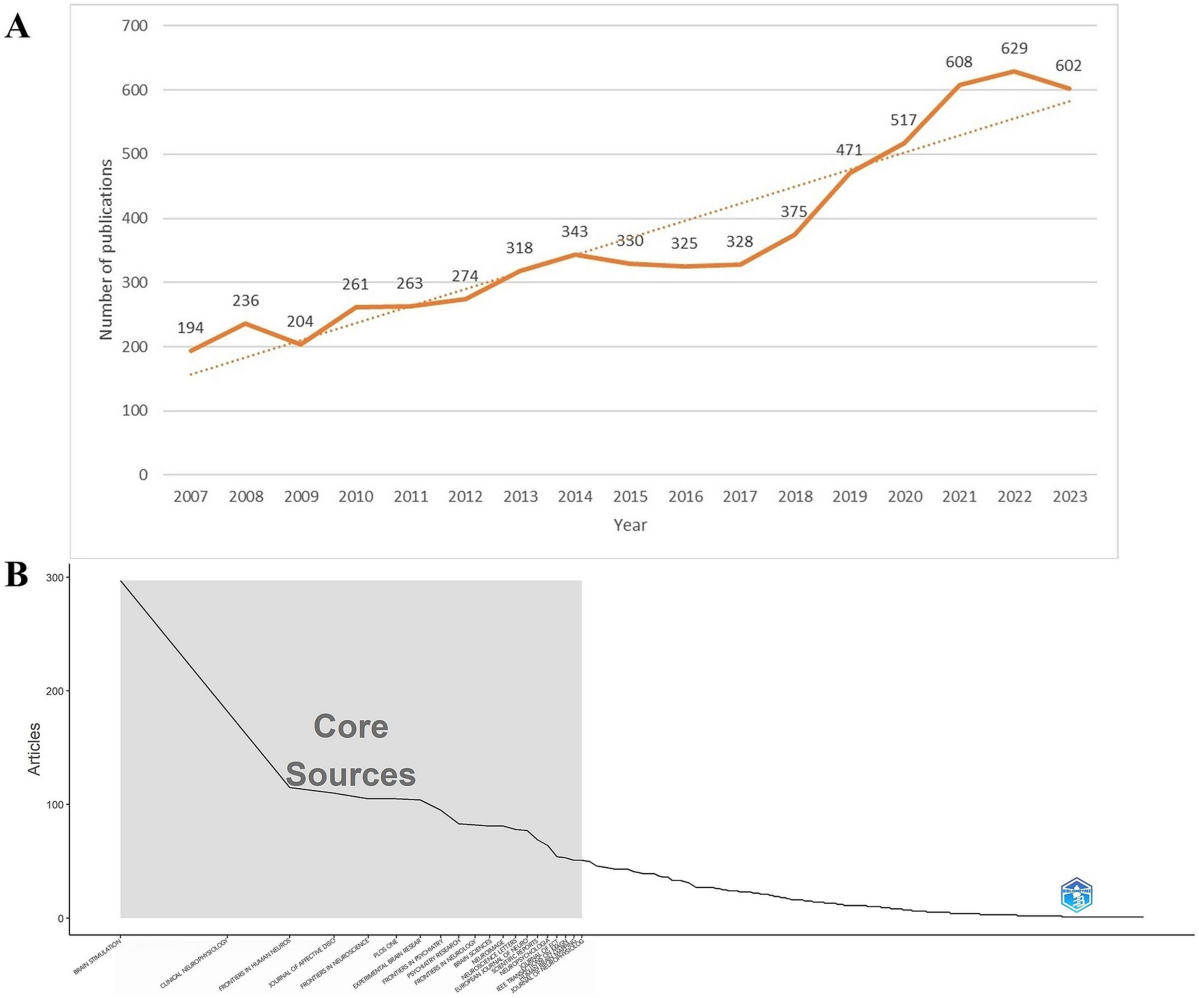
**(A)** Annual publication products of relevant TMS articles from 2004 to 2023. TMS, Transcranial magnetic stimulation. **(B)** The top 20 journals are core journals.

A total of 9,100 TMS-related documents were initially retrieved. After restricting the document types to articles and reviews and limiting the language to English, 6,278 publications remained, comprising 5,485 articles and 793 reviews. Figure 2A illustrates the annual publication output (0 publications in the period 2004–2006). During 2007–2017, the number of articles showed a slow rise with erratic and slightly fluctuating growth. The period 2018–2023 showed a rapid and steady growth. The highest growth in the number of articles was seen in the period 2009–2010 with 57 articles. The number of publications exhibited a decline in 2008–2009 and 2014–2016. The number of articles exceeded 300 after 2013. The number of articles increased by more than 90 per year from 2018 to 2019 and from 2020 to 2021, and the number of articles exceeded 600 in 2021. Table 1 shows the 10 major funding sources. A total of 4,655 articles on TMS were funded by major funding. The top three funding sources were the United States Department of Health and Human Services, National Institutes of Health, and National Natural Science Foundation of China.

**Table 1:** The top 10 funding sources.

Rank	Funding agencies	Frequency	Countries	Percentage of total articles
1	United States Department of Health Human Services	634	USA	10.099
2	National Institutes of Health	633	USA	10.083
3	National Natural Science Foundation of China	482	China	7.678
4	National Health Medical Research Council of Australia	185	Australia	2.947
5	Ministry of Education Culture Sports Science and Technology Japan	174	Japan	2.772
6	Canadian Institutes of Health Research	169	Canadian	2.692
7	Japan Society for The Promotion of Science	163	Japan	2.596
8	National Institute of Mental Health	161	USA	2.565
9	German Research Foundation Deutsche Forschungsgemeinschaft	156	German	2.485
10	UK Research Innovation	143	UK	2.278

### 3.2 Journal analysis

Between 2004 and 2023, a total of 786 journals reported research literature on TMS. The top 10 journals with the most published literature are shown in Table 2. The publication output of these journals accounts for 20.71% of the total number of publications. The journal ranked first in terms of publication volume contributes to 5.05% of the total publications. Eight of the top 10 journals focused on TMS-related neuroscience aspects and two on psychiatry. Among them, the most active journal is *Brain Stimulation*, which has published 317 articles. It also has the highest H-index (23) and ranks second in terms of average citations per article (44.78). *Clinical Neurophysiology* ranks second in both publication output (199 articles) and H-index (24), while it ranks first in terms of average citations per article (78.57). The *Journal of Affective Disorders* ranks third in terms of publication output with 122 articles. Both *Brain Stimulation* and *Journal of Affective Disorders* have an average citation per article exceeding 20.

**Table 2:** Top 10 journals with the most publications.

Rank	Journal	Publications	No. of times cited	No. of times cited (per article)	H-index	G-index	Citation density	IF (2023)	JCR
1	Brain Stimulation	317	14,196	44.78	62	96	4.93	7.6	Clinical Neurology Q1; Neurosciences Q1
2	Clinical Neurophysiology	199	15,636	78.57	54	119	7.38	3.7	Clinical Neurology Q1; Neurosciences Q2
3	Journal of Affective Disorders	122	3,133	25.68	35	47	3.64	4.9	Clinical Neurology Q1; Psychiatry Q1; Psychiatry Q1
4	Frontiers in Human Neuroscience	121	1963	16.22	23	39	2.14	2.4	Neurosciences Q3; Psychology Q2
5	Frontiers in Neuroscience	120	1,362	11.35	21	28	2.33	3.2	Neurosciences Q2
6	Plos One	119	3,281	27.57	35	49	3.77	2.9	Multidisciplinary Sciences Q1
7	Experimental Brain Research	106	3,501	33.03	33	55	2.54	1.7	Neurosciences Q4
8	Frontiers in Psychiatry	103	935	9.08	15	24	2.06	3.2	Psychiatry Q2; Psychiatry Q2
9	Frontiers in Neurology	93	1,231	13.24	19	27	2.92	2.7	Clinical Neurology Q2; Neurosciences Q3
10	Neuroscience Letters	92	1749	19.01	23	34	1.77	2.5	Neurosciences Q3

The G-index and impact factor of the journals are shown in Table 2. *Brain Stimulation* was ranked first in G-index (96) and also had the highest impact factor (7.6). *Journal of Affective Disorders* is ranked second with an impact factor of 4.9. There are five journals with IF > 3, namely *Brain Stimulation*, *Clinical Neurophysiology*, *Journal of Affective Disorders*, *Frontiers in Neuroscience*, and *Frontiers in Psychiatry*. According to the journal citation report, there are four journals located in the Q1 region, namely *Clinical Neurology*, *Neurosciences*, *Psychiatry*, and *Multidisciplinary Science*. In Figure 2B, the top 20 journals are core journals according to Bradford’s Law. Core journals have received a great deal of attention and great popularity among researchers and can provide important guidance and references for researchers to publish journals.

### 3.3 Co-occurrence analysis

#### 3.3.1 Author collaboration analysis

**Figure 3:**
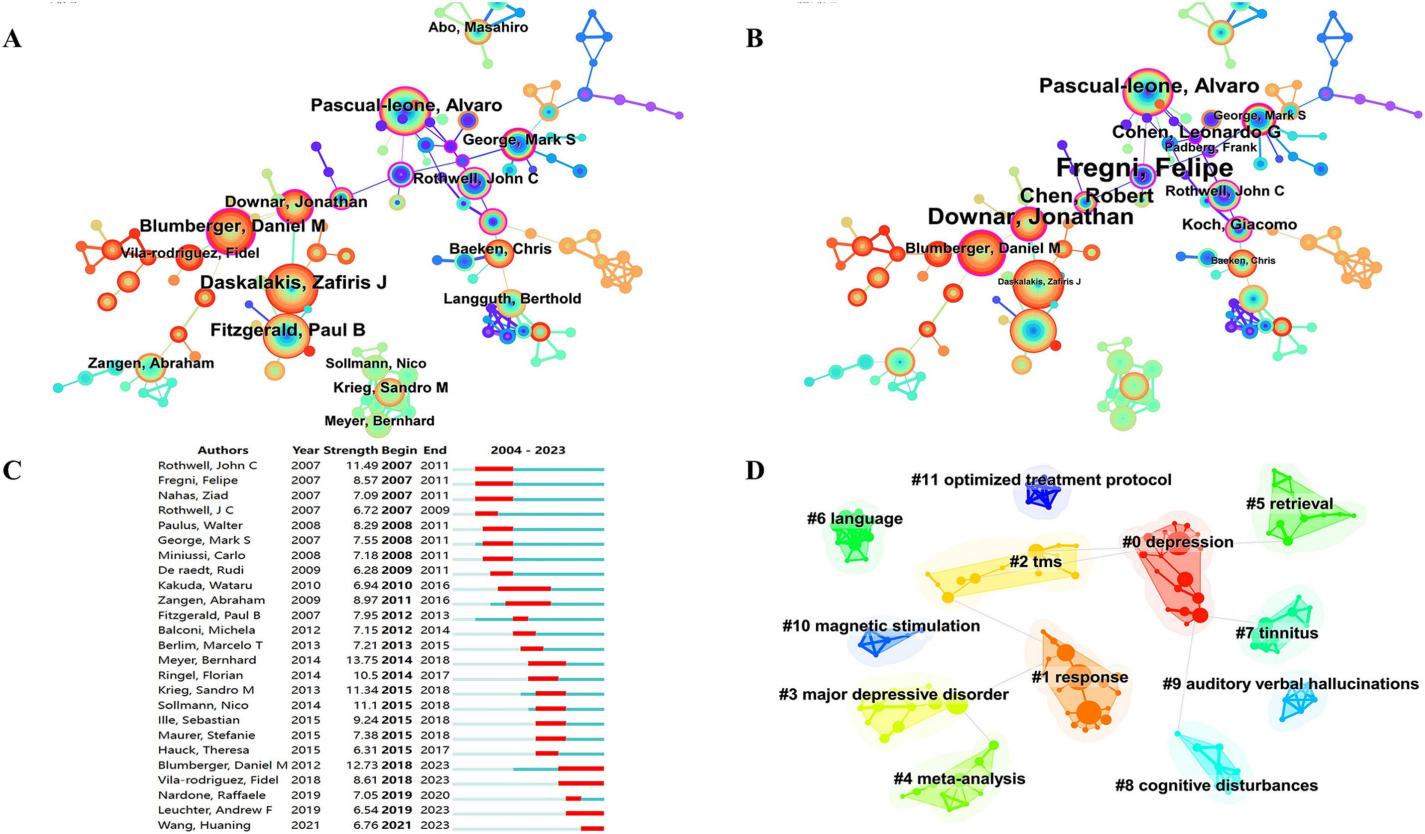
Co-occurrence map of authors. **(A)** Top 15 authors in citation counts. **(B)** Top 12 authors in centrality. **(C)** Top 25 strongest bursts authors. **(D)** Author clustering map.

The 6,278 papers we retrieved were published by a total of 19,113 authors. Table 3 shows the top 10 authors with the highest number of publications. The top three authors were Daskalakis ZJ from University of Toronto (166), Fitzgerald PB from Monash University (134) and Pascual-Ieone A from Hebrew SeniorLife (129). The author Pascual-leone A has the highest H-index (25), number of citations (13,228) and G-index (90). Fitzgerald PB has the second highest G-index (26). Most of the top authors are from Canada and the United States. Figure 3 presents the co-occurrence map of co-authors. Daskalakis ZJ has collaborated with Fitzgerald PB from Monash University and Blumberger DM from the University of Toronto. These individuals collectively form key institutional intermediaries within the co-authorship network. Their collaboration has been particularly active in the research on TMS treatment for depression. Within the same institution, the connection between Daskalakis ZJ and Blumberger DM is the most frequent. Additionally, Blumberger DM from the University of Toronto has collaborated with Downar J and Vila-Rodriguez F. Across countries and institutions, Daskalakis ZJ and Fitzgerald PB have maintained a close collaboration. This has formed a core cluster centered around Daskalakis ZJ. Pascual-leone A formed an international collaboration with Rothwell JC (University College London) and George MS (Medical University of South Carolina). Figure 3B shows that the top 3 authors according to centrality are Fregni F, Downar J, and Pascual-leone A, respectively. Authors with strong outbreaks can be identified based on Figure 3C. Meyer, Bernhard has the strongest outbreaks (13.75), with outbreaks from 2014 to 2018. Second is Blumberger, DM with outbreaks from 2018 to 2023. Third is Rothwell, John C with outbreaks from 2007 to 2011. Means that the number of articles published by these authors in the relevant TMS increases rapidly over a certain period of time. Figure 3D forms 12 clusters. Cluster labels represent keywords in the co-occurrence network. Cluster 0 contains the most keywords, including depression, Hf-rTMS, attention, rTMS, TBS.

**Table 3:** Top 10 authors with the most publications.

Rank	Authors	Institutions	Publications	No. of times cited	No. of times cited (per article)	Citation density	H-index	G-index	Percentage of total articles
1	Daskalakis ZJ	University of Toronto	166	8,745	52.68	6.24	52	75	2.644
2	Fitzgerald PB	Monash University	134	7,387	55.13	5.44	48	76	2.134
3	Pascual-leone A	Harvard Medical School	129	13,228	102.54	8.36	56	90	2.055
4	Blumberger DM	University of Toronto	100	3,935	39.35	6.31	33	55	1.593
5	Rothwell JC	University College London	81	8,907	109.96	8.67	45	67	1.29
6	George MS	Medical University of South Carolina	74	10,450	141.22	11.1	39	60	1.179
7	Langguth B	University of Regensburg	74	4,593	62.07	7.39	30	58	1.179
8	Baeken C	Ghent University	70	4,571	65.3	8.1	29	65	1.115
9	Zangen A	Ben Gurion University	66	7,909	119.83	10.01	35	59	1.051
10	Downar J	University of Toronto	63	3,472	55.11	8.13	30	54	1.004

#### 3.3.2 Institutional cooperation analysis

**Figure 4:**
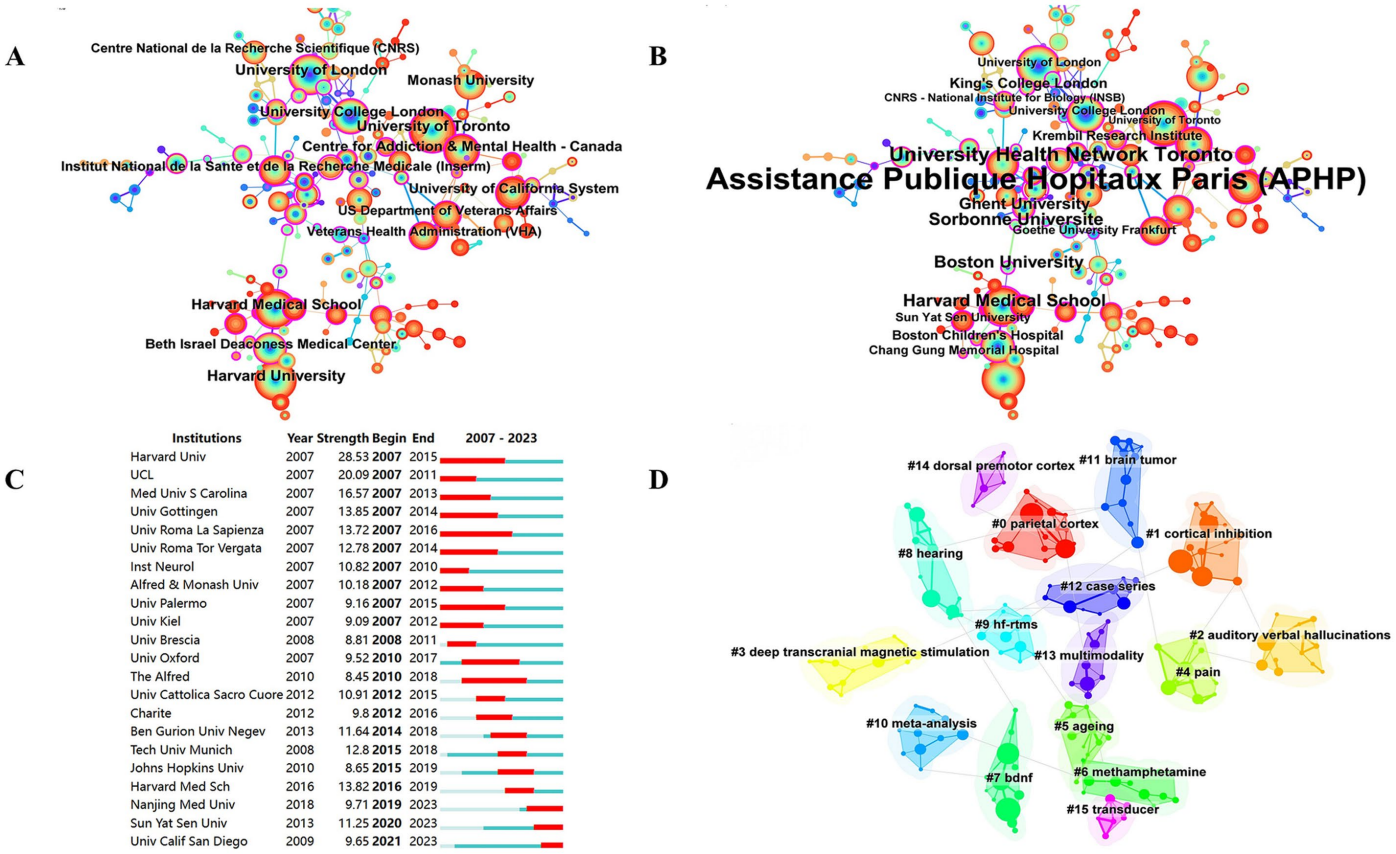
Co-occurrence map of institutions. **(A)** Top 13 institutions in citation counts. **(B)** Top 16 institutions in centrality. **(C)** Top 22 strongest bursts institutions. **(D)** A clustering map of the network of institutions.

Between 2004 and 2023, a total of 6,278 studies related to TMS were published by 4,242 major institutions. The top 10 institutions are shown in Table 4. Harvard University from the United States leads the other institutions at the top of the list with 292 publications, a total of 18,527 citations, and the highest H-index (27). This was followed by University of Toronto (285) from Canada and University of London (258) from the UK. Figure 4 depicts the largest sub-network between cooperating organizations constructed by Citespace. The top 3 by centrality are APHP from France, University Health Network Toronto from Canada, and Harvard Medical School from the United States. Most of these top-ranking organizations are from the United States and Canada, and their outstanding achievements are inextricably linked to strong national policy and funding support. Domestically, Harvard University collaborates with Harvard Medical School, University of California System, and Beth Israel Deaconess Medical Center. Among these collaborations, Harvard Medical School is the organization with the most publications and the most citations. In Figure 4C, the top 3 institutions with strong outbreaks are Harvard Univ, UCL, and Med Univ S Carolina, with the outbreaks all starting in 2007. In Figure 4D, there are 16 cluster labels in the institutions. The results of the analysis of the institutional clustering map reveal key patterns of collaboration and knowledge flows in academic research. This visualization tool allows us to observe the clustering of different research institutions within specific research areas. For example, the study of the clinical efficacy of TMS on hearing, vision, balance, and movement forms a dense area, highlighting the research hotspots and intensity of collaboration within these areas.

**Table 4:** Top 10 institutions.

Rank	Affiliations	Publications	Countries	No. of times cited	No. of times cited (per article)	H-index	Citation density	Percentage of total articles
1	Harvard University	292	USA	18,527	63.45	66	6.00	1.465
2	University of Toronto	285	Canadian	17,860	62.67	64	6.75	2.596
3	University of London	258	UK	19,686	76.3	62	6.09	1.258
4	Harvard Medical School	226	USA	15,793	69.88	62	6.53	3.138
5	University College London	201	UK	17,944	89.27	60	6.92	1.911
6	Centre For Addiction Mental Health Canada	197	Canada	10,102	51.28	55	6.16	1.322
7	University of California System	178	USA	6,920	38.88	44	4.61	1.418
8	Monash University	173	Australia	8,333	48.17	50	5.05	3.6
9	Beth Israel Deaconess Medical Center	163	USA	14,496	88.93	62	7.30	4.651
10	Institut National De La Sante Et De La Recherche Medicale	160	France	7,644	47.78	40	6.01	2.549

#### 3.3.3 Country cooperation analysis

**Figure 5:**
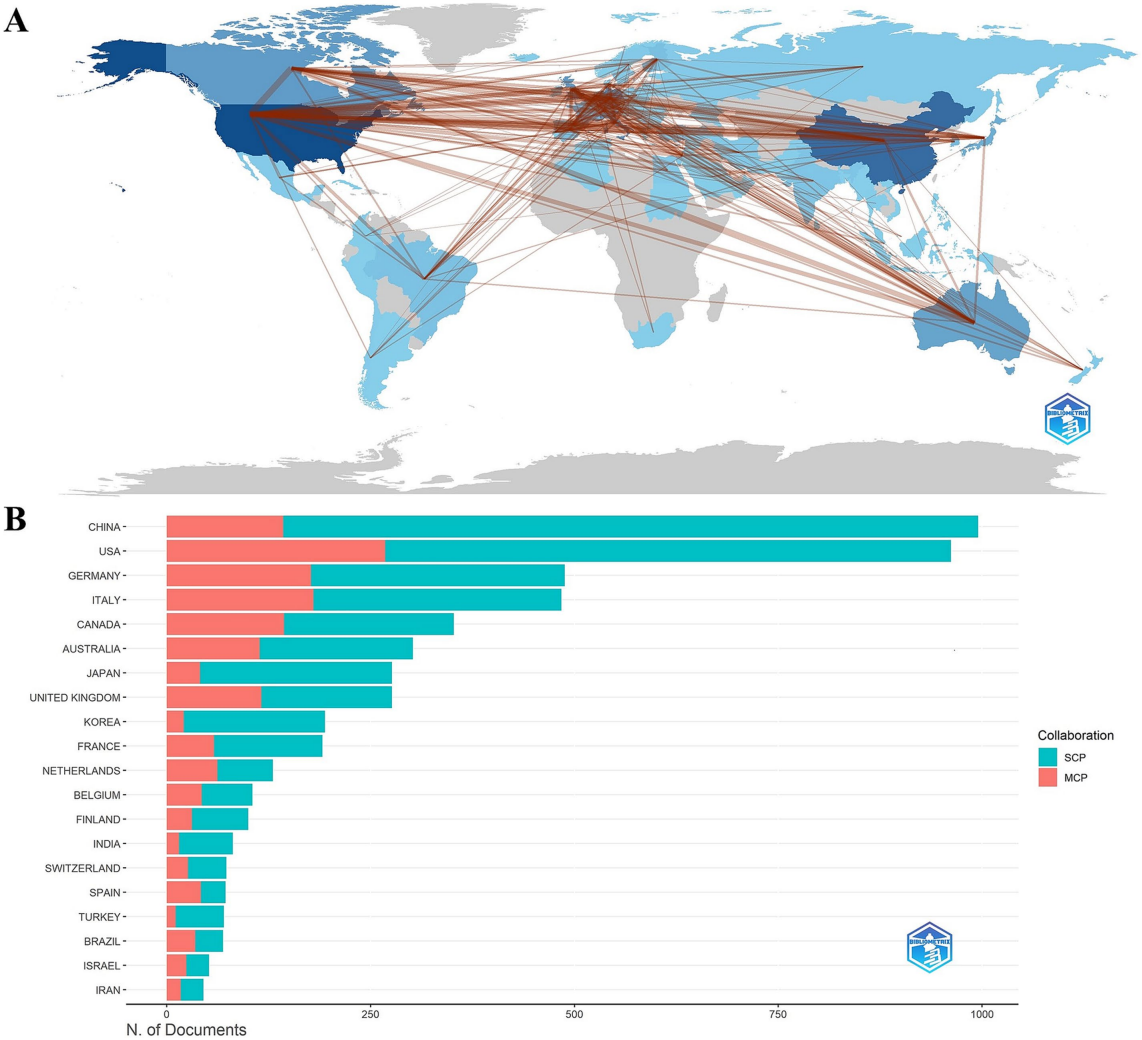
**(A)** Country collaboration map. **(B)** Cooperation network map of countries. SCP, Single Country Publications; MCP, Multiple Country Publications.

**Figure 6:**
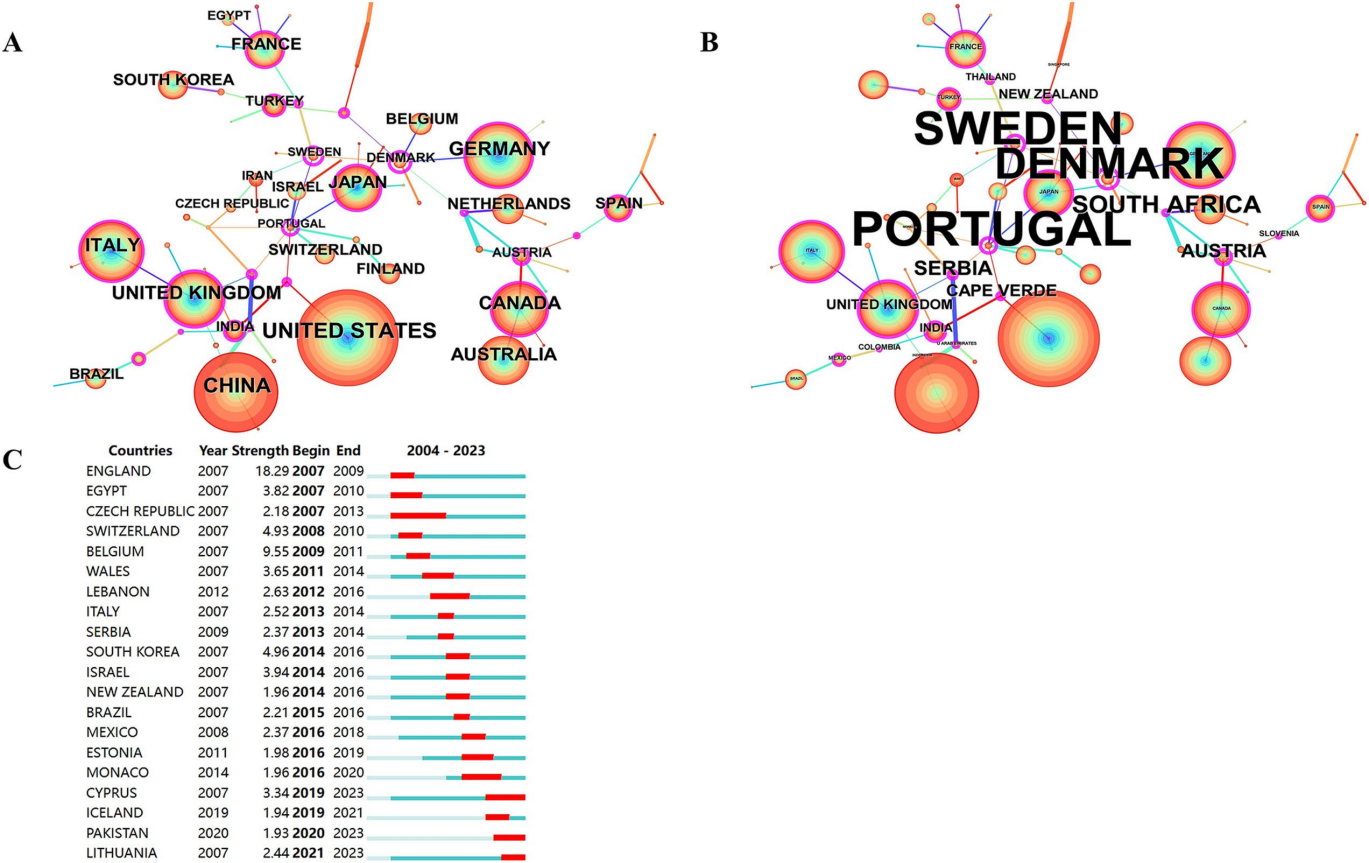
Co-occurrence analysis of countries. **(A)** Countries ranked by citation counts. **(B)** Countries ranked by centrality. **(C)** Top 20 strongest bursts countries.

A total of 85 countries were involved in the publication of relevant TMS studies. The top 10 countries are shown in Table 5. The United States ranks first with 1,528 publications and a total of 61,671 citations. It was followed by China (1074), Germany (701) and Italy (664). Figure 5A illustrates a map of country cooperation. Extensive international cooperation exists in TMS, and as seen in Figure 5B, the USA has the most collaborative publications with other countries (MCP: 268), followed by Italy (MCP: 180), Germany (MCP: 177), Canada (MCP: 144), and China (MCP: 143). Proportion of international article cooperation Canada (MCP proportion: 40.9%), Italy (MCP proportion: 37.2%), Germany (MCP proportion: 36.3%), the United States (MCP proportion: 27.9%), and China (MCP proportion: 14.4%). The USA cooperates primarily with Canada (frequency of cooperation: 135), Italy (frequency of cooperation: 114), China (frequency of cooperation: 104), the United Kingdom (frequency of cooperation: 95), and Australia (frequency of cooperation: 92). The United States had the most publications in international cooperation, while the most independent publications were submitted by China (SCP:852). Figures 6A,B presents a co-presentation diagram between the collaborating countries. Portugal, Sweden and Denmark lead the centrality rankings in bibliometrics, and they play a crucial role in the area of cooperation in TMS-related literature, facilitating the exchange of information between countries. China cooperates with the United States, Italy, and the United Kingdom, with the United States predominating. Japan has cooperation with Switzerland, Portugal, and Israel. Figure 6C, the top 3 countries in the strong explosiveness rankings are England, Belgium, and South Korea. Suggesting that these countries have significantly increased their research output in a short period of time, and may have a great potential for development in the future.

**Table 5:** Top 10 countries in publications from 2004 to 2023.

Rank	Countries	Publications	No. of times cited	No. of times cited (per article)	H-index	Citation density	Percentage of total articles
1	USA	1,528	61,671	40.36	104	4.25	24.339
2	China	1,074	16,586	15.44	60	2.72	17.107
3	Germany	701	32,768	46.74	80	26.94	11.166
4	Italy	664	31,705	47.75	77	25.96	10.577
5	Canada	580	25,991	44.81	74	22.40	9.239
6	England	542	29,834	55.04	81	22.12	8.633
7	Australia	478	19,053	39.86	69	15.01	7.614
8	Japan	354	13,620	38.47	48	16.50	5.639
9	France	296	16,494	55.72	56	21.48	4.715
10	South Korea	231	4,486	19.42	39	5.71	3.68

### 3.4 Reference co-citation analysis

**Figure 7:**
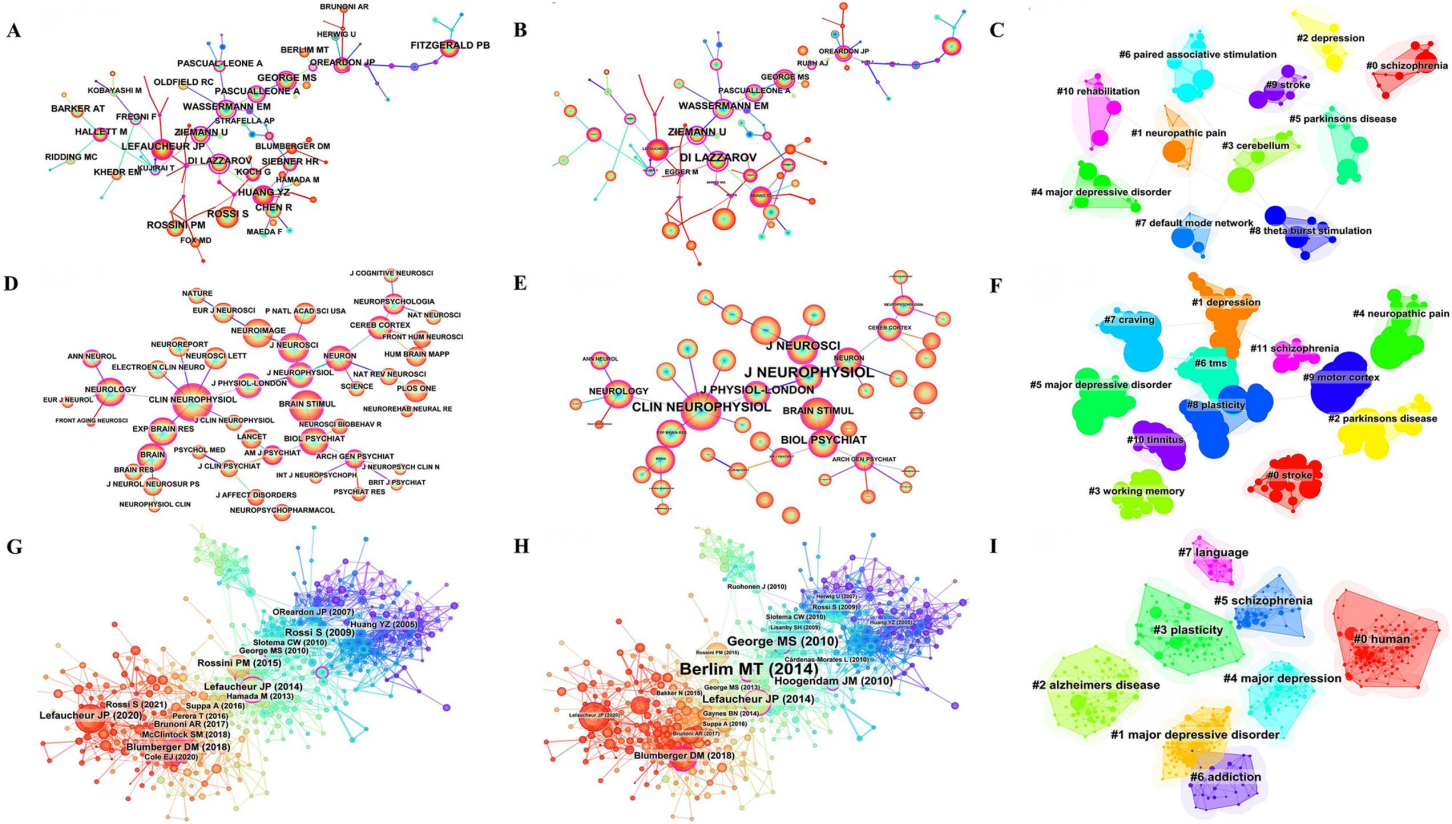
Co-citation analysis of authors, journals, references. **(A)** Network map of co-cited authors by citation counts. **(B)** Network map of co-cited authors by centrality. **(C)** Clustering map of co-cited authors. **(D)** Network map of co-cited journals by citation counts. **(E)** Network map of co-cited journals by centrality. **(F)** Clustering map of co-cited journals. **(G)** Network map of co-cited reference by citation counts. **(H)** Network map of co-cited reference by centrality. **(I)** Co-citation reference clustering map.

Table 6 shows the top 10 co-cited references. Helping us to understand the research base of TMS related fields may be used to assess the degree of relationship between the literatures. The top-ranked co-cited literature was cited a total of 3,804 times, with an average of 237.75 co-citations per year. This is followed by a seminal article published in 2014 by Lefaucheur, J.P. et al. in *Clinical Neurophysiology*, a prominent journal in the field. Another publication authored by Lefaucheur, J.P. et al. ranks second in terms of average annual citations (212.40). The third most cited work overall is a study by O’Reardon, J.P. et al., published in *Biological Psychiatry*. Notably, The *Lancet* maintains the highest impact factor (98.4) among these journals. Figure 7 illustrates the co-citation analysis of authors, journals, and references. Figures 7A–C present the author co-citation network and cluster visualization, respectively. The analysis reveals that Lefaucheur, J.P., Rossi S., Huang Y.Z., and Di Lazzaro V. rank among the top authors in terms of co-citation frequency. Notably, a co-citation relationship exists between Lefaucheur, J.P. and Fregni F. Furthermore, Wassermann E.M., Ziemann U., and Di Lazzaro V. demonstrate high centrality measures within the network. Figures 7D–F display the journal co-citation network and corresponding cluster visualization. Our analysis identifies the top three journals by co-citation as *Clinical Neurophysiology*, *Brain Stimulation*, and *NeuroImage*. In terms of centrality measures, the leading journals are *Journal of Neurophysiology*, *Clinical Neurophysiology*, and *Journal of Neuroscience*. Figures 7G–I present the reference co-citation subnetworks and cluster visualization, respectively. The analysis reveals eight distinct cluster labels: Human, Major Depressive Disorder, Alzheimer’s Disease, Plasticity, Major Depression, Schizophrenia, Addiction, and Language.

**Table 6:** The top 10 reference with most co-citation counts.

Rank	Title	First authors	Journal	IF (2023)	Publication year	Total citations	Average per year
1	Safety, ethical considerations, and application guidelines for the use of transcranial magnetic stimulation in clinical practice and research	Rossi, S	Clinical Neurophysiology	3.7	2009	3,804	237.75
2	Evidence-based guidelines on the therapeutic use of repetitive transcranial magnetic stimulation (rTMS)	Lefaucheur, JP	Clinical Neurophysiology	3.7	2014	1,356	123.27
3	Efficacy and safety of transcranial magnetic stimulation in the acute treatment of major depression: A multisite randomized controlled trial	O’Reardon, JP	Biological Psychiatry	9.6	2007	1,225	68.06
4	Transcranial magnetic stimulation: A primer	Hallett, M	Neuron	14.7	2007	1,189	66.06
5	Evidence-based guidelines on the therapeutic use of repetitive transcranial magnetic stimulation (rTMS): An update (2014–2018)	Lefaucheur, JP	Clinical Neurophysiology	3.7	2020	1,062	212.40
6	A practical guide to diagnostic transcranial magnetic stimulation: Report of an IFCN committee	Groppa, S	Clinical Neurophysiology	3.7	2012	848	65.23
7	Daily Left Prefrontal Transcranial Magnetic Stimulation Therapy for Major Depressive Disorder A Sham-Controlled Randomized Trial	George, MS	Archives of General Psychiatry	14.5	2010	724	48.27
8	Efficacy of Transcranial Magnetic Stimulation Targets for Depression Is Related to Intrinsic Functional Connectivity with the Subgenual Cingulate	Fox, MD	Biological Psychiatry	9.6	2012	709	54.54
9	Effectiveness of theta burst versus high-frequency repetitive transcranial magnetic stimulation in patients with depression (THREE-D): a randomized non-inferiority trial	Blumberger, DM	Lancet	98.4	2018	659	94.14
10	Technology Insight: noninvasive brain stimulation in neurology - perspectives on the therapeutic potential of rTMS and tDCS	Fregni, F	Nature Clinical Practice Neurology	7.6	2007	624	34.67

rTMS, Repetitive transcranial magnetic stimulation; tDCS, Transcranial direct current stimulation.

### 3.5 Keyword co-occurrence analysis

**Figure 8:**
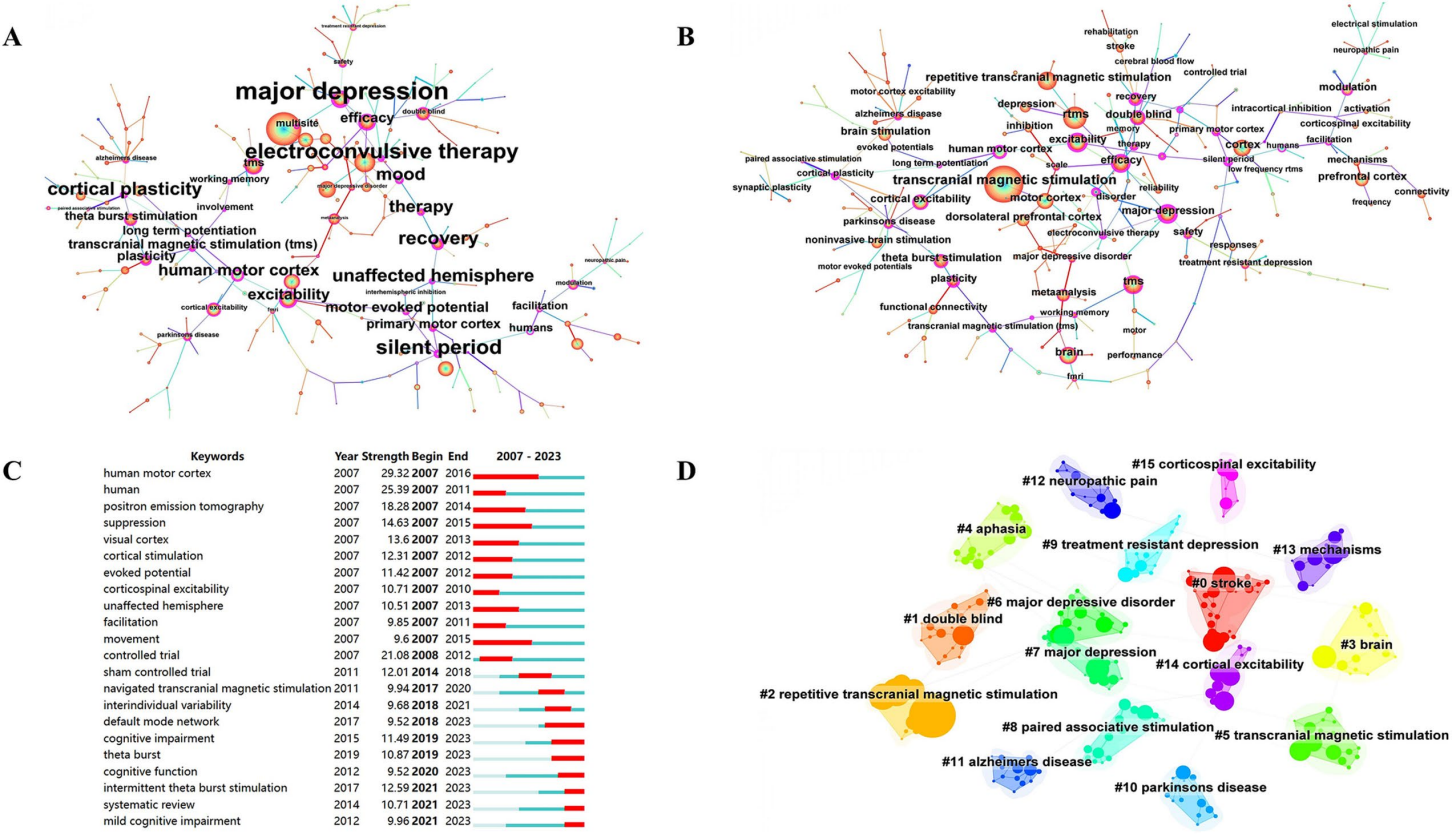
Co-occurrence map of keywords. **(A)** Keywords ranked by citation counts. **(B)** Keywords ranked by centrality. **(C)** Top 22 strongest bursts keywords. **(D)** A clustering map of the co-citation network of keywords.

**Figure 9:**
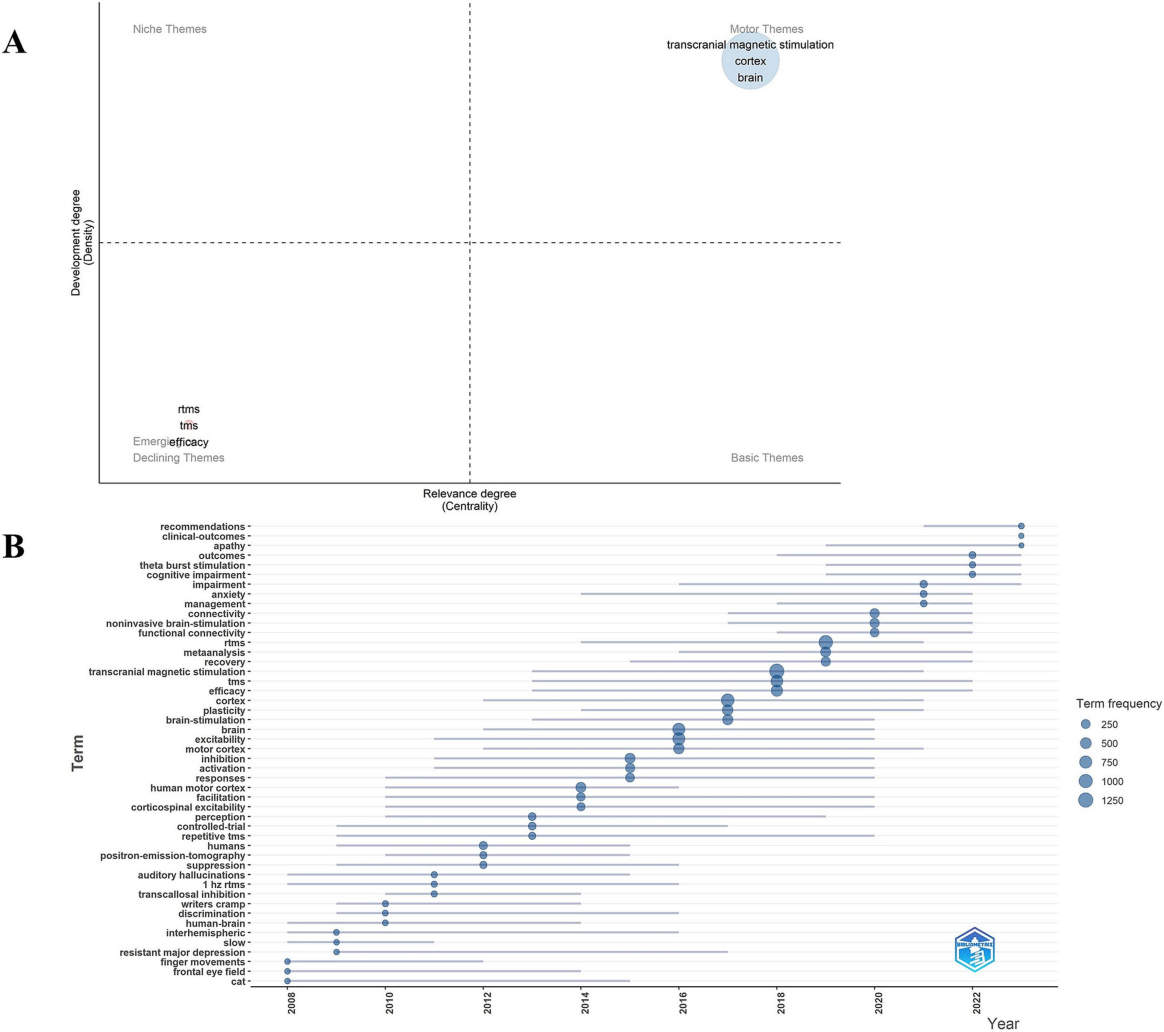
**(A)** Keyword theme map. **(B)** Trend chart of keyword emergent terminology changes.

Co-occurrence analysis of keywords has been mainly employed to obtain cutting-edge knowledge and research trends in related TMS. Figure 8 presents the co-occurrence map of the keywords. In Figure 8A, the most common keywords ranked by co-occurrence count were motor cortex, brain, efficacy, excitability, double blind, theta burst stimulation, major depression, dorsolateral prefrontal cortex. Figure 8B, the top 3 ranked by centrality are major depression, electroconvulsive therapy, silent period. Figure 8C, the top 3 keywords ranked by strong explosiveness are human motor cortex, human, controlled trial. Figure 8D, gives an idea about the cluster analysis of the keywords. The keyword clustering can be divided into the following categories: diseases, such as stroke, aphasia, major depressive disorder, major depression, treatment resistant to depression, Parkinson’s disease, Alzheimer’s disease, neuropathic pain; TMS categories, such as TMS, rTMS, paired-associative stimuli; and a category that may involve TMS mechanisms, such as cortical excitability, corticospinal excitability, mechanisms. There are 16 clusters (Supplementary Table S1 for detailed results of keyword clustering) with Q value of 0.8555 and silhouette value of 0.9686. Figure 9A depicts the keyword topic map. TMS, cortex, and brain are in the upper right corner, indicating that they are important and well developed. Figure 9B. Trends in subject terminology. Research themes in 2008 included finger movements, frontal eye field, and cat. More frequent in recent years have been connectivity, noninvasive brain-stimulation, and functional connectivity. The most frequent occurrences were TMS (1279) in 2018, rTMS (1062) in 2019, cortex (861) in 2017, brain (747) in 2016, and excitability (739) in 2016.

## 4 Discussion

We conducted bibliometric and visual analyses of TMS-related literature retrieved from the WOSCC using two analytical tools: CiteSpace and R language. Our investigation revealed a consistent upward trend in publication output from 2004 to 2023. The most productive and influential author is Daskalakis ZJ with 166 publications and 8,745 citations. The journals *Brain Stimulation* and *Clinical Neurophysiology* have demonstrated the highest productivity in terms of article output and citation frequency. Among research institutions and countries, Harvard University and the United States have emerged as the most active contributors in this field. Research institutions and investigators from the United States, Canada, and the United Kingdom constitute the core research consortium in the TMS field, maintaining extensive and robust international collaborations. The most frequently occurring keywords include motor cortex, brain, efficacy, excitability, double-blind, theta-burst stimulation, major depression, and dorsolateral prefrontal cortex. Recent emerging hotspots in the field are reflected by the following keywords: intermittent theta-burst stimulation, cognitive impairment, systematic review, and mild cognitive impairment.

### 4.1 Publication output

Over the past two decades, TMS-related research publications have demonstrated a consistent upward trajectory, a finding that aligns with the results reported by Zheng et al. (28). In their study. The accelerated growth observed since 2018 may be attributed to the limited efficacy of conventional therapeutic approaches for various dysfunctions caused by central nervous system injuries (such as stroke, spinal cord injury, and Parkinson’s disease) and associated pain complications, while rTMS has demonstrated promising therapeutic outcomes (29, 30). The publication output has exhibited a rapid increase, which is consistent with previous research findings. For instance, the integration of fMRI-guided target selection in TMS has enabled more precise modulation of brain functional networks (31), offering novel approaches for clinical TMS localization. The advancement of therapeutic technologies has significantly propelled TMS-related research, which has also been substantially supported by national funding initiatives.

### 4.2 Journals

These journals encompass diverse research domains including neuroscience, psychiatry, and psychological/affective studies, thereby providing valuable reference points for researchers in selecting appropriate publication venues. The core journals predominantly originate from the United States, Switzerland, and the Netherlands. Among the top 10 journals, most have published over 100 articles, with impact factors consistently exceeding 3.0. These journals are primarily classified within the Q1 category, indicating their reliability and prominence in TMS-related research. The citation counts of articles vary significantly across different journals. The Irish journal *Clinical Neurophysiology* has accumulated the highest total citation count (15,636). Among these, the most cited publication is an Expert Guidelines article that updates safety protocols for TMS research and clinical applications (32), while also addressing safety considerations for novel stimulation devices. Among the top 10 journals, *Frontiers in Psychiatry* has recorded the lowest total citation count (935). Notably, one of its valuable contributions includes a study demonstrating comparable therapeutic efficacy between Hf-rTMS over the left-DLPFC and Lf-rTMS applied to the contralateral homologous region in treating major depressive disorder (33).

### 4.3 Scientific collaboration network

Through co-occurrence analysis of authors, institutions, and countries, we can delineate the global collaborative network in TMS research. The top-ranked authors are exclusively affiliated with European institutions. According to Lotka’s Law and Price’s Law (34, 35), researchers with more than 10 publications are identified as core authors. The analysis reveals a total of 445 core authors, among whom the top 10 contributors include three researchers from the University of Toronto (Canada), two from the United States, and one each from the United Kingdom, Australia, and Germany.

The most prolific contributor is Daskalakis Z.J. from the University of Toronto, with a total of 8,745 citations. As a leading researcher in the field, he has made substantial contributions to the advancement of TMS applications in depression treatment. He has demonstrated that bilateral TBS shows superior efficacy compared to standard rTMS in treating treatment-resistant depression (TRD) among elderly patients (36). Furthermore, his research indicates that the differences in clinical outcomes among various rTMS protocols for acute major depressive episodes are negligible (37), and that intermittent TBS (iTBS) exhibits comparable therapeutic effects to Hf-rTMS (38). Additionally, cortical plasticity in the DLPFC can be assessed through the combination of repetitive paired associative stimulation (rPAS) with EEG (39). Notably, Fitzgerald P.B. from Monash University and Downar J. from the University of Toronto have collaborated to develop an MRI-guided approach for optimal TMS coil positioning over the DLPFC (40). This MRI-TMS collaboration represents an innovation in TMS. Given the involvement of the DLPFC in various psychiatric disorders, this methodology facilitates precise regional brain stimulation, thereby enabling targeted therapeutic interventions (41). These developments provide valuable guidance for clinical practice. Pascual-Leone A from Harvard Medical School and Zangen A have extensively collaborated on animal studies, investigating the complementary effects of Hf-rTMS and lorazepam in suppressing epileptic seizures in rats. EEG recordings revealed that the efficacy of Hf-rTMS remained unaffected by lorazepam dosage (42). However, rTMS is not currently recommended for epilepsy treatment in clinical settings, which underscores the need for further investigation into the relationship between TMS and epilepsy. Their research also revealed that Lf-rTMS significantly reduces motor cortex excitability through long-term depression mechanisms, as validated in anesthetized rat models (43). These findings provide fundamental evidence for understanding the inhibitory effects of Lf-rTMS on cortical excitability.

The top 10 institutions are all from Western countries, which have abundant resources, advanced experimental equipment, and excellent research teams. Harvard University, University of Toronto, University of London, Harvard Medical School, and University College London account for 65.41% of the total publications among the top 10 institutions. They represent the core research forces and major contributors in this field, indicating that the United States, Canada, and the United Kingdom may lead the global research on TMS. Daskalakis ZJ, Blumberger DM, and Downar J have formed intra-institutional collaborations, and they found that rTMS did not demonstrate positive therapeutic effects in patients with schizophrenia treated with clozapine (44). This appears to be inconsistent with the conclusion that rTMS can alleviate symptoms in patients with schizophrenia (45). The specific influencing factors warrant further investigation. Harvard University and Harvard Medical School maintain a robust domestic institutional collaboration, having co-authored 292 publications, among which 7 have achieved remarkable citation counts. The University of Toronto and the University of London have established an international partnership, jointly developing evidence-based guidelines for rTMS treatment protocols (46). In 2007, they collaborated and combined NIRS with TMS, discovering that the level of hemoglobin in the motor cortex increased following TMS (47). In 2013, they developed a plan to investigate the changes in hemoglobin concentration after sp-TMS (48). This indicates that the NIRS-TMS system is gradually maturing, and the substantial progress in imaging technology has promoted the development of TMS.

Among the top 10 countries with the highest publication output, 70% are from the European region, while 30% are from the Asian region, which includes China, Japan, and South Korea. It is evident that China has made significant contributions in the Asian region. However, there are no Asian countries among the top-ranked authors and institutions. Differences in the overall economic and scientific and technological development of a country may be one of the key reasons contributing to regional disparities in TMS-related scientific research output (49, 50). Notably, most of the top ranked countries in TMS research output are developed nations. The theoretical basis, hardware equipment, and technical practice of TMS originated in Western countries and have since been disseminated globally. For example, the electromagnetic theory was proposed in Western countries, and the initial magnetic stimulation device emerged in the UK (51); in 1995, researchers in the USA were delved into the effects of TMS on depressed mood (52); in 2008, the FDA approved the first TMS device for the clinical treatment of depression (53). In contrast, in China, TMS equipment, particularly advanced TMS with navigation and localization capabilities is available in only a limited number of hospitals or research institutes, primarily due to its relatively high cost.

Financial support is also one of the factors affecting the research output, and it correlates with a nation’s overall economic and technological standing. Notably, three out of the top 10 funding sources are from the US, which underpins a remarkable 22.75% of TMS research. Funding from Western countries accounts for a substantial proportion. Among Asian nations, China ranks second in publication volume, just after the US, while Japan stands at 8th place in terms of output. This is likely closely connected to the national financial backing (Among the top 10 funding sources, those from China and Japan supported 7.678 and 5.368%, respectively, of the total publications). We observe that Germany and Canada, ranking highly in funding, also achieve prominence in article output (3rd and 5th places, respectively). The UK demonstrates exceptional strength, ranking 2st in H-index despite being 6th in total output. Moreover, research output correlates with the number of researchers. In the TMS research field, the United States and China rank first and second, with 5,899 and 5,465 researchers respectively, accounting for 28.081 and 26.015% of all authors. In addition, several other factors can influence TMS research output and impact. These include safety regulations for emerging diagnostic and therapeutic technologies in the field, ethical approvals for clinical/animal studies, and healthcare payment methods (54). The limited adoption of costly, out-of-pocket treatments may also affect clinical research (55). In China, rTMS was initially self-funded, which has restricted its clinical application.

In terms of international collaboration, Canada has the highest proportion. It has a close working relationship with the United States, with a total of 1,529 articles published jointly. This indicates that European countries still dominate the field of TMS research. China, with 1,075 independent publications, stands out as the country with the highest number of single-country publications. This suggests that there is significant room for China to enhance its international collaboration and exchange, presenting substantial opportunities for growth in global partnerships.

The most highly co-cited article is by Rossi S et al., published in *Clinical Neurophysiology*, with an average annual citation count of 237.75. In this study, the authors evaluated the safety of TMS in clinical applications (32). Building on traditional TMS protocols, patterned repetitive TMS has been developed, with technological advancements facilitating the design of novel devices. These innovations have enabled the real-time integration of TMS with electroencephalography (EEG), positron emission tomography (PET), and functional magnetic resonance imaging (fMRI) (56). This progress provides an objective foundation for the clinical application of TMS as a therapeutic intervention. In 2014, Lefaucheur JP et al. established guidelines for the use of rTMS in the early stages of various conditions, including pain, stroke, and schizophrenia, providing a reliable evidence-based foundation for the clinical application of rTMS (46). In 2020, he updated these guidelines, further emphasizing the role of rTMS in the treatment of neuropathic pain and depression (57). The most highly co-cited article, with an impact factor of 98.4, is a randomized, multicenter clinical trial published in the *Lancet* in 2018. This study compared the efficacy of iTBS with that of Hf-rTMS in treating depression, demonstrating that iTBS achieves satisfactory outcomes in patients with treatment-resistant major depressive disorder (38). The growing body of evidence underscores the feasibility and significance of TMS as a therapeutic intervention for neurological and psychiatric disorders.

*Clinical Neurophysiology* ranks first in total citations (4,653) and second in centrality (0.52). Serving as a key connecting node, *Clinical Neurophysiology* has fostered close collaborations among journals such as *Neurology*, *Experimental Brain Research*, *Journal of Physiology*, *Neuroscience Letters*, *NeuroReport*, *Electroencephalography* and *Clinical Neurophysiology*, and *Journal of Clinical Neurophysiology*. Most of these collaborative efforts originate from researchers based in the United States and the Netherlands. Clinical neurophysiology encompasses the study of cerebral neurophysiology, which may help elucidate the mechanisms underlying the effects of TMS. Notably, a high journal impact factor tends to attract more high-quality submissions. *Journal of Neurophysiology* ranks first in centrality and has established collaborations with journals such as *Journal of Neuroscience*, *Journal of Physiology*, *Neuron*, and *Brain Stimulation*, with the most prominent collaborations being with *Journal of Neuroscience* and *Journal of Physiology*. These collaborations are primarily driven by contributions from the United States. This highlights the significant role of *Journal of Neurophysiology* in the field of TMS research, facilitating the dissemination of knowledge related to TMS.

### 4.4 Keywords

According to existing data, the keywords are associated with motor evoked potentials, parietal cortex, theta-burst stimulation, major depression, dorsolateral prefrontal cortex, stroke, double-blind studies, and neuropathic pain, outcomes, clinical-outcomes, meta-analysis. As a diagnostic tool, the combined application of TMS with various imaging techniques, such as fMRI and functional near-infrared spectroscopy (fNIRS), provides a powerful approach for neuroscience research and clinical treatment. This integration enables precise localization of stimulation targets, optimization of treatment parameters, and offers a robust foundation for the diagnosis and treatment of neurological disorders. On the other hand, TMS exerts its therapeutic effects by modulating multiple pathways, including glial cell polarization, synaptic remodeling, and neurotransmitter systems. It has demonstrated significant efficacy and broad application potential in the treatment of conditions such as neuropathic pain, depression, and stroke.

The integration of TMS with imaging technologies has emerged as a significant direction in both neuroscience research and clinical applications, representing a major research focus in the TMS field. This convergence provides a novel perspective for exploring brain function and the mechanisms underlying neurological disorders. The MEP module of TMS can accurately reflect the excitability of the cerebral cortex. Studies have shown that MEP amplitude is positively correlated with enhanced functional connectivity in the brain (58), making it a powerful indicator for assessing the prognosis of patients with stroke or spinal cord injuries. In addition, the combined application of TMS-EEG enables real-time monitoring of cortical excitability changes. For instance, Casarotto et al. utilized TMS-EEG to observe alterations in Parkinson’s disease (PD) patients following levodopa treatment, revealing a significant increase in cortical excitability in the motor regions (24). In patients with schizophrenia, TMS of the frontal cortex resulted in a reduced gamma-band response in EEG (59), suggesting potential inhibition of neuronal activity. Similarly, the combined application of TMS and fMRI has become increasingly prevalent, providing a precise tool for assessing the modulation of brain functional connectivity networks. For example, fMRI studies in post-stroke aphasia patients have shown weakened functional connectivity in the affected hemisphere (25). In patients with borderline personality disorder, rTMS of the right-DLPFC revealed reduced connectivity between the amygdala, insula, and posterior default mode network nodes on fMRI (60). Additionally, Hf-rTMS (10 Hz) significantly enhanced activation in the affected motor cortex (M1) of stroke patients, while Lf-rTMS reduced activation in the contralateral hemisphere (61), suggesting that Hf-rTMS offers greater advantages in improving motor function. The combination of fNIRS and TMS has opened new avenues for neuroscience research. fNIRS offers advantages such as portability and resistance to motion artifacts, making it more practical for clinical applications. For instance, when Hf-rTMS was applied to different brain regions (M1, S1, PMC, PFC) in patients with neuropathic pain following spinal cord injury, fNIRS detected suppressed activation in M1 and PMC, accompanied by significant pain relief (62). This integration not only enhances the precision of target selection but also provides robust support for the clinical translation of TMS. The target point is a key parameter in rTMS therapy, and precise localization has become a major focus in the recent development of rTMS technology. A number of studies indicate that neuronavigation can facilitate accurate brain-area localization during rTMS, enhancing treatment efficacy (63). Moreover, neuronavigation-guided rTMS can assist in evaluating surgical-resection margins when used alongside surgery (64). Looking ahead, integrating TMS with emerging techniques like fMRI, fNIRS, neuronavigation-based localization, and EEG may hold the key to achieving more precise TMS-based neuromodulation.

Keyword clustering analysis suggested depression stroke aphasia Parkinson’s disease Alzheimer’s disease and neuropathic pain are important areas of TMS research. This is highly consistent with clinical practice. For instance HF-rTMS applied to the DLPFC has been demonstrated to significantly alleviate depressive symptoms (23). Similarly in stroke rehabilitation rTMS can improve post-stroke swallowing function limb spasticity and cognitive performance (65–67). Studies indicate that rTMS enhances phonological naming ability in aphasic patients including improvements in noun retrieval and verbal output. Furthermore rTMS may modulate dopamine release in specific subregions of the medial prefrontal cortex suggesting potential therapeutic benefits for Parkinson’s disease symptoms (27, 68). Furthermore its cognitive-enhancing effects in patients with mild-to-moderate Alzheimer’s disease persist for up to three months (69). Significant progress has also been made in applying rTMS to neuropathic pain management with evidence-based medical support established for M1 region stimulation in treating neuropathic pain (46, 70). Current research extends to exploration of alternative targets including the DLPFC and parietal lobe (71, 72). In clinical applications rTMS has primarily been used for neuropsychiatric disorders but its potential in musculoskeletal conditions is now being explored. Novel rTMS protocols for postmenopausal osteoporosis have been proposed (73) and preliminary studies on its application in knee osteoarthritis have been conducted (74). New discoveries in these areas can be anticipated in the future.

rTMS is widely used in clinical practice, with stimulation frequency, target location, treatment duration, and stimulation protocols varying across different disorders. The optimal paradigms for specific diseases remain under active investigation. The common keywords “outcomes, clinical outcomes, meta-analysis” and the keyword cluster #1 “double-blind” suggest that clinical randomized controlled studies or meta-analyses aimed at providing evidence-based evidence for each TMS treatment are an important area of TMS-related research. The therapeutic target of TMS is the topic of interest. The cerebellum has emerged as a novel target for rTMS, demonstrating significant potential in treating movement disorders, psychiatric conditions, and neurorehabilitation. rTMS application to the cerebellum can markedly improve motor ataxia symptoms in patients with hereditary ataxia by modulating the hyperactive cerebello-thalamo-cortical pathway (43). Furthermore, cerebellar rTMS combined with language training has been shown to enhance language recovery in post-stroke patients (75). In psychiatric applications, cerebellar stimulation has exhibited therapeutic effects in alleviating depressive symptoms and reducing negative symptoms in schizophrenia patients (76). The stimulation frequency of TMS represents a critical determinant of its clinical efficacy, with parameter selection being equally essential in therapeutic applications. For instance, Hf-rTMS applied to the lesioned hemisphere or low-frequency rTMS to the non-lesioned hemisphere has been shown to significantly improve memory function in patients with mild cognitive impairment (26). A double-blind randomized controlled trial demonstrated that Lf-rTMS combined with conventional speech therapy can reduce barriers to functional reorganization in aphasia patients (77). Meta-analytic evidence indicates that Hf-rTMS provides significantly greater benefits for motor function in Parkinson’s disease compared to low-frequency protocols (78). The same high-frequency approach has also proven effective in ameliorating obsessive-compulsive disorder symptoms and shows moderate-to-strong evidence for treating schizophrenia symptoms (68, 79). With advancing research, novel TMS paradigms have emerged. Notably, iTBS offers shorter treatment durations than conventional rTMS while demonstrating temporal therapeutic advantages in achieving rapid early improvement of depressive symptoms (80).

Key Term Cluster #13 highlights “mechanisms” as a major research focus, with several underlying pathways currently identified. For instance, TMS exerts its effects through several pathways, including modulating glial cell-mediated neuroinflammation, influencing synaptic plasticity, and regulating mitochondrial function. Studies have demonstrated that Hf-rTMS alleviates neuropathic pain through multiple mechanisms, such as downregulates neuronal nitric oxide synthase (nNOS) expression, suppresses astrocyte activation and proliferation, and modulates neuroinflammation (81). Notably, Hf-rTMS achieves significant analgesic effects by both reducing pro-inflammatory cytokines (e.g., IL-1β and IL-6) and regulating neurotransmitter systems (particularly glutamate and dopamine) (70). Besides, rTMS enhances the anti-inflammatory effects of microglia by inhibiting NF-κB and signal transducer and activator of transcription 6 (STAT6) (82). Hf-rTMS suppresses microglial M1 polarization via the let-7b-5p/HMGA2/NF-κB signaling pathway (83). Modulating synaptic plasticity is another key mechanism of rTMS, and the specific molecular pathways involved may include regulating brain-derived neurotrophic factor (BDNF) gene expression, increasing synaptic protein markers such as synaptophysin, and enhancing the expression of Ca(2+)/calmodulin-dependent protein kinase II (CaMKII) (84, 85). Studied found that TMS also modulates mitochondrial function. rTMS improves symptoms in patients with bipolar disorder by restoring the functional state of mitochondria, producing ATP, and mediating actin-mitochondrial cross-linking (86). Alternatively, TMS reduces mitochondrial degeneration in rats (87). Some other potential mechanisms included the affections on neurotransmitter systems, neural networks, histone lactylation, methylation, and modulation of ion channels. For example, rTMS can reverse depressive behaviors by increasing histone and DNA methylation (88). rTMS may upregulate ion channels such as Na(+) channels, A-type K(+) channels, and Ca(2+) channels to increase the excitability of hippocampal neurons (89).

## 5 Conclusion

We conducted a comprehensive analysis of TMS-related literature from 2004 to 2023, revealing the dynamic growth and evolving trends in this field. The United States leads globally in TMS research output, with Harvard University standing out as the most active institution. Brain Stimulation, Clinical Neurophysiology, and Journal of Affective Disorders are the most influential journals. Daskalakis Z.J. (Canada) has demonstrated the highest productivity, forming key collaborations with Fitzgerald P.B. and Blumberger D.M. While TMS research benefits from extensive global collaboration, further cooperation among leading authors, institutions, and nations is still required to drive innovation and knowledge exchange. Current research hotspots include TMS’s integration with imaging techniques like fNIRS, EEG, and fMRI, its applications in clinical diseases, optimization of diagnostic and therapeutic parameters, exploration of evidence-based applying, and investigation of potential neurological modulation mechanisms. In the future, continued innovation and collaboration promise to unlock new possibilities for TMS applications in both research and clinical practice.

## 6 Limitation

This study has the following limitations. The first one is that we only analyzed the literature related to TMS in WOSCC. Although relevant literature is available in Scopus and PubMed, we exclude it. The reason for this is the unavailability of citation reports from PubMed, and the possibility of duplication of information as well as loss of information when merging different databases is taken into account. Hence, we ended up choosing WOSCC, which qualifies core journals and generates citation reports on various aspects to get more detailed content. The second is that the publication years for our analysis are 2004–2023, and there are a number of recently published studies that were not involved and may have overlooked the value of relevant research.

## Data Availability

The original contributions presented in the study are included in the article/Supplementary material, further inquiries can be directed to the corresponding author.
